# Assessment of a laboratory critical risk result notification protocol in a tertiary care hospital and their use in clinical decision making

**DOI:** 10.11613/BM.2019.030703

**Published:** 2019-08-05

**Authors:** Jose A. Delgado Rodríguez, Maria I. Pastor García, Cristina Gómez Cobo, Antonia R. Pons Más, Isabel Llompart Alabern, Josep Miquel Bauça

**Affiliations:** 1 Department of Laboratory Medicine, Hospital Universitari Son Espases, Palma, Spain; 2 Instituto de Investigación Sanitaria de las Islas Baleares, Spain

**Keywords:** phone notification, turnaround time, clinical decision making, critical risk result

## Abstract

**Introduction:**

Communication of laboratory critical risk results is essential for patient safety, as it allows early decision making. Our aims were: 1) to retrospectively evaluate the current protocol for telephone notification of critical risk results in terms of rates, efficiency and recipient satisfaction, 2) to assess their use in clinical decision making and 3) to suggest alternative tools for a better assessment of notification protocols.

**Materials and methods:**

The biochemical critical risk result notifications reported during 12 months by routine and STAT laboratories in a tertiary care hospital were reviewed. Total number of reports, time for the notification and main magnitudes with critical risk results were calculated. The use of notifications in clinical decision making was assessed by reviewing medical records. Satisfaction with the notification protocol was assessed through an online questionnaire to requesting physicians and nurses.

**Results:**

Critical result was yielded by 0.1% of total laboratory tests. Median time for notification was 3.2 min (STAT) and 16.9 min (routine). The magnitudes with a greater number of critical results were glucose and potassium for routine analyses, and troponin, sodium for STAT. Most notifications were not reflected in the medical records. Overall mean satisfaction with the protocol was 4.2/5.

**Conclusion:**

The results obtained indicate that the current protocol is appropriate. Nevertheless, there are some limitations that hamper the evaluation of the impact on clinical decision making. Alternatives were proposed for a proper and precise evaluation.

## Introduction

A significant percentage of clinical decisions rely on laboratory test results (*1*). In this context, great efforts have been invested in improving the preanalytical and analytical phase, since most situations that pose patient safety risk lie in these phases (*2*, *3*). The next step for laboratories needs to address postanalytical issues, that is, the way laboratory test results are reported to the requesting physician and the quickness of this information (*4*, *5*).

Clinical laboratories often obtain unexpected results requiring immediate medical attention and action because they indicate a high risk of imminent death or major patient harm, also called critical risk results (*6*, *7*).

Notification of critical risk results to the physician by telephone has been shown to reduce the time needed for diagnosis and initiation of treatment, hence decreasing the associated morbidity and mortality. Therefore, a proper strategy for critical risk result notification, rather than an obligation for clinical laboratories, is both a right for the patient in order to ensure safety and a crucial point for healthcare systems, which reduces costs and saves lives (*8*).

In this regard, the ISO 15189:2012 accreditation requires clinical laboratories to design and establish a protocol for a critical risk results and a periodic evaluation of the system. Without universal consensus or objective evidence, it is a major challenge for laboratories to create and maintain their own alert list. In order to promote “best practice” and harmonization of critical risk results management, guidelines and recommendations have been published, most recently by the Clinical and Laboratory Standards Institute (CLSI), the Australasian Association of Clinical Biochemists and the Royal College of Pathologists (*9*-*11*). Critical risk result notification protocols need to be a dynamic process under continuous improvement (*12*). For this reason, indicators are required for the monitoring of the whole process. Consensus among physicians and laboratory professionals is essential for the definition of such indicators and their review (*13*, *14*).

Different working groups have suggested indicators for this evaluation, including the Working Group on Laboratory Errors and Patient Safety of the International Federation of Clinical Chemistry and Laboratory Medicine (IFCC LEPS) (*15*). From a clinical perspective, there is scarce literature regarding indicators to assess clinical impact, such as those suggested by Lopez *et al.* (*16*). Hence, comparisons between findings in different hospitals are challenging (*17*).

The aims of our study were: 1) to retrospectively evaluate the current protocol for telephone notification of critical risk results at a tertiary care hospital in terms of rates, efficiency and recipient satisfaction, 2) to assess the use of these notifications in clinical decision making by the attending physicians and 3) based on these, to suggest alternative tools for a better assessment of notification protocols.

## Materials and methods

### Study design

This is a retrospective observational study performed at the Hospital Universitari Son Espases (Palma de Mallorca, Spain), providing service to a population of 325,285 inhabitants. The biochemistry core laboratory gives support to the specialization units of the hospital, the primary care centres, as well as to other institutions (penitentiary facilities, county hospitals, *etc.*) and is accredited in accordance with ISO 15189:2012.

In this study an evaluation was made of the protocol for critical risk result notification by telephone, which has been valid since 2012.

### Notification protocol

The magnitudes and their critical risk results were established through consensus between laboratory and medical personnel based on medical literature and taking into consideration the fact that the hospital is a tertiary care centre, the prevalence of diseases in our region and the medical specialties available (*17*, *18*). The definitive alert list is outlined on Table 1. Critical risk criteria were the same for routine and STAT laboratory analyses. Haematology and coagulation-related magnitudes were excluded due to the different protocol for notification, performed by other specialists.

**Table 1:** Critical results requiring phone notification

**Analyte (unit)**	Critical results**
**General biochemistry**	
Calcium (mmol/L)	< 1.6 or > 3.2
Chloride (mmol/L)	< 75 or > 120
Ethanol (mmol/L)	> 0.9
Glucose (mmol/L)	< 2.2 or > 25
Magnesium (mmol/L)	< 0.6 or > 2.1 (adults); < 0.4 (children)
Phosphate (mmol/L)	< 0.3 or > 2.9
Potassium (mmol/L)	< 2.5 or > 6.5
Sodium (mmol/L)	< 125 or > 160
Troponin I (ng/L)*	> 80 (women); > 170 (men)
Ionized calcium (mmol/L)	< 0.8 or > 1.6
**Blood gas analysis**	
pH	≤ 7.20 or ≥ 7.60
pCO_2_ (kPa)	< 2.7 or > 9.3 (arterial)
pO_2_ (kPa)	< 6.0 (arterial)
Bicarbonate (mmol/L)	< 10 or > 40 (arterial)
**Therapeutic drug monitoring**	
Acetaminophen (µmol/L)	> 123
Carbamazepine (µmol/L)	> 85
Cyclosporine (nmol/L)	> 500
Digoxin (nmol/L)	> 2.8
Lithium (mmol/L)	> 2.0
Phenytoin (µmol/L)	> 79
Phenobarbital (µmol/L)	> 172
Salicylate (mmol/L)	> 2.9
Theophylline (µmol/L)	> 222
Valproate (µmol/L)	> 693
*Only emergency room is notified. **The established critical values were based on agreement between physicians and laboratory professionals.	

The protocol for critical risk result is as follows: when a critical risk result is obtained in the laboratory, and confirmed by repetition, the laboratory information system (LIS, GestLab, Indra, Spain) identifies it and a notification request is automatically created (critical result flag). After ruling out preanalytical interferences (haemolysis, diluted sample, sample contamination with anticoagulant, *etc*.), result notification is performed telephonically by the laboratory staff (specialist in clinical biochemistry or laboratory technician) to the attending professional (physician or nurse), and immediately the LIS notification request is filled with the univocal identification of the professional receiving the notification. Likewise, the personal code of the professional who makes the notification is also recorded on the LIS. The notified test is highlighted on the laboratory report. Therefore, a total traceability throughout the whole notification process is assured. Results are subsequently validated and a laboratory report is generated. The lack of timeframes for reporting is consistent with all hospital departments or with primary care centres and critical result notification is carried out by telephone only. As escalation procedure, if the critical result applies to an outpatient and the requesting physician could not be notified, the emergency healthcare service is contacted through a 061-call (emergency number in Spain).

### Evaluation of turnaround time (TAT)

For this evaluation, information stored in the LIS was used with regard to critical risk result notifications reported between January and December 2017.

Efficiency of the notification strategy was assessed by means of quality indicators in the different medical areas: percentage of critical risk result notifications with respect to the total number of test results in that area, time required for notification (TAT: defined as the period between the apparition of a critical risk result on the LIS after repetition and the moment when the notification request is filled with the code of the professional who receives the notification), and percentage of unsuccessful notifications (defined as the impossibility of notifying a critical risk result after several failed attempts).

The percentage of critical risk result notifications by magnitude was quantified and related with the most prevalent pathologies attended at our hospital. A single notification may imply several critical results (*e.g.* a single patient sample showed high glucose concentration together with high potassium).

Turnaround time notifications were classified according to the CLSI GP47 guideline into: ‘timely’ if below 15 minutes, ‘acceptable’ if between 15 and 60 minutes, and ‘inappropriate’ if above 60 minutes (*9*).

### Satisfaction with critical result notification protocol

An online survey was prepared in order to find out to what extent attending physicians and nurses were satisfied with different aspects of the laboratory, including accessibility, kindness, incidence communication, quality of laboratory reports, *etc.* (unpublished data). One of the questions referred to the professional’s satisfaction with the current critical result notification protocol in our hospital. The survey was prepared on SurveyMonkey.com and internally circulated by email to all attending professionals in our healthcare area between June to December 2018. Participation was voluntary and anonymous. Satisfaction scoring established between 0 (very bad) and 5 (excellent).

Surveyed universe was all attending physicians (1014) and nurses (1496) in our hospital, as well as in primary care centres. Data were downloaded on Microsoft Excel 2010 (Microsoft, USA).

### Evaluation of the use of notifications in clinical decision making

In accordance with the communication channels in our clinical laboratory, assessment of notification strategy usefulness was achieved by randomly reviewing 100 medical records from patients at the emergency room (ER), along with the medical records of all patients from the outpatient consultation offices in our hospital who had have at least one critical result from the laboratory within the evaluated period. The following were quantified as effectiveness indicators:

1. Percentage of notifications made which appeared in the medical records of the patient by the total number of critical risk result notifications reviewed by department.
2. Percentage of cases where physicians carried out corrective actions: requested inter-specialty consulting, requested complementary testing, initiated or changed treatments, or (if outpatients) derived them to the hospital after laboratory notification with respect to the total number of medical record reviewed by department.

### Statistical analysis

The Kolmogorov-Smirnov test was used to assess normality of all variables. Differences between groups were assessed using the Student’s t-test if normally distributed, and the Mann-Whitney’s U-test if not. Statistical significance was set at 0.05. SPSS v.24 software (IBM Corporation, USA) was used for all statistical analyses.

## Results

Table 2 shows the percentages of successful and failed notifications of critical risk results in the different medical areas, as well as turnaround times (medians and interquartile ranges) and their classification according to the CLSI GP47 guideline. When assessing the differences between notification turnaround times for the different medical areas from our routine laboratory by means of the Mann-Whitney’s U-test, statistically significant differences were found between hospitalized and primary care patients (P = 0.019) and between hospitalized and outpatients (P = 0.004). Likewise, there was a greater response rate for notifications made from the stat laboratory, with significant differences with any of the routine laboratory subgroups (P < 0.001).

**Table 2:** Results and classification of the notifications

		**Total number of results**	Critical results*, **N (%)**	Notified critical results**, ** N (%)**	**Unsuccessful notifications^#^, N (%)**	**Turnaround time^§^ (min)**	**Classification^$^**
**Routine** **testing**	Primary care	2.807,822	170 (< 0.01)	156 (92)	0	19 (10-34)	Acceptable
	Hospitalized	936,167	1407 (0.15)	583 (41)	0	15 (8-28)	Acceptable
	Outpatients	750,293	136 (0.02)	96 (71)	1 (0.7)	21 (10-41)	Acceptable
	Other	264,333	92 (0.03)	71 (77)	0	18 (11-34)	Acceptable
	Total	4.758,615	1805 (0.04)	906 (50)	1 (0.1)	17 (9-31)	Acceptable
**STAT testing**	Emergency Room	646,706	3796 (0.59)	3212 (85)	13 (0.3)	3 (2-7)	Timely
**Total**		5.405,321	5601 (0.10)	4118 (74)	14 (0.3)	4 (2-12)	Timely
*The percentage of critical results was calculated with respect to the total number of results in that area. **The percentage of notified critical results was calculated with respect to the number of critical results detected by the laboratory information system. ^#^The percentage of unsuccessful notifications was calculated with respect to the number of critical results detected by the laboratory information system. ^§^Turnaround time is presented as median and interquartile range of the time between the apparition of a critical notification request in the laboratory information system and its filling with the univocal identification of the professional receiving the notification. ^$^The classification of turnaround time according to reference 9.							

Percentages of critical risk result notifications are outlined in Table 3, according to the different magnitudes (notifications for ionized calcium, chloride and ethanol were not presented since they accounted for < 0.01% of all notifications). The group defined as ‘Other’ includes notifications not defined previously, but which were made under professional discretion (considering previous results known for patient, medical records or any other criteria), such as a creatinine or alanine aminotransferase (ALT) value that had significantly increased in a short period of time (*e.g.* creatinine from normality to 600 µmol/L, or ALT from normality to 3000 U/L).

**Table 3:** Size of critical results regarding analyte and respective department notified by phone during the assessment

	**Routine testing**					**STAT testing**	**Total** **(N = 4118)**
**Analyte**	**Primary Care** **(N = 156)**	**Hospitalization** **(N = 583)**	**Outpatients** **(N = 96)**	**Other** **(N = 71)**	**Total Routine** **(N = 906)**	**(N = 3212)**
Calcium	6 (4%)	38 (7%)	5 (5%)	6 (9%)	55 (6%)	121 (4%)	176 (4%)
Phosphate	4 (3%)	61 (11%)	13 (14%)	2 (3%)	80 (9%)	48 (1%)	128 (3%)
Glucose	69 (44%)	129 (22%)	24 (25%)	21 (30%)	243 (27%)	511 (16%)	754 (18%)
Magnesium	3 (2%)	53 (9%)	7 (7%)	3 (4%)	66 (7%)	97 (3%)	163 (4%)
Potassium	44 (28%)	136 (23%)	34 (35%)	17 (24%)	231 (26%)	401 (13%)	632 (15%)
Sodium	21 (14%)	134 (23%)	6 (6%)	22 (31%)	183 (20%)	596 (19%)	779 (19%)
Troponin I	-	-	-	-	-	738 (23%)	738 (18%)
Blood gas analysis	0 (0%)	22 (4%)	6 (6%)	0 (0%)	28 (3%)	573 (18%)	601 (15%)
Therapeutic drug monitoring	0 (0%)	4 (0.7%)	0 (0%)	0 (0%)	4 (0.4%)	93 (3%)	97 (2%)
Other	9 (6%)	6 (1%)	1 (1%)	0 (0%)	16 (2%)	34 (1%)	50 (1%)

Regarding the online questionnaire circulated to physicians and nurses in our healthcare area, a response rate of 7.9% was achieved for hospital professionals. Results are highlighted in Table 4.

**Table 4:** Questionnaire for attending physicians and nurses on the critical result notification protocol

**Workplace**	**Professional category**	**Answers,** **N (%)**	**Satisfaction degree, N (ratio)**					
			**0**	**1**	**2**	**3**	**4**	**5**
H. Universitari Son Espases	Physician (specialist/resident)	88 (30)	0	0	0	9 (0.10)	36 (0.41)	43 (0.49)
	Nurse	102 (34)	0	7 (0.07)	10 (0.10)	16 (0.16)	34 (0.33)	35 (0.34)
Primary Care center	Physician (specialist/resident)	72 (24)	0	0	1 (0.01)	2 (0.03)	17 (0.24)	52 (0.72)
	Nurse	36 (12)	0	0	1 (0.03)	5 (0.14)	19 (0.53)	11 (0.30)
Total		298 (100)	0	7 (0.02)	12 (0.04)	32 (0.11)	106 (0.36)	141 (0.47)
The satisfaction degree was asked to be graded between 0 (very bad) to 5 (excellent).								

With regard to the use of notifications in clinical decision making, results are shown in Table 5.

**Table 5:** Quantification of clinical actions reflected in the medical records reviewed after critical result notification, depending on patient’s origin

**Effectiveness indicators**		**Critical results for the ER patients (N = 100)**	**Critical results for the outpatients (N = 86)**
Notification stated at medical record		6	21
	Initiation/change treatment	51	31
	Admission/ Complementary testing	43	-
**Corrective actions**	Referred to ER	-	17
	Inter-consultation	6	2
	Biochemistry testing repeated	0	4
	**Total**	100	54
ER - emergency room.			

## Discussion

### Critical values, TAT and departments

Evaluation of the communication system for critical risk result notification in our laboratory shows that the total number of notifications meet the international recommendations, although lower than the 0.4% obtained in the study performed by Arbiol and colleagues (*12*, *19*). This could be due to the fact that blood gas point-of-care testing analysers are distributed throughout our hospital (ER, intensive care unit, obstetrics unit, *etc.*), and their critical results do not appear in the LIS. Our alert list also differs significantly from those reported elsewhere, including the low threshold for sodium ion or the high threshold for potassium ion (*20*). Although adoption of those values was considered in our hospital, the current protocol is based on its satisfactory results and in order to avoid excessive telephone calls (especially by lowering the high potassium ion threshold from 6.5 mmol/L to 6.0 mmol/L).

Notified critical risk results for primary care are also in agreement with the values found in literature (4.0-20.5%) (*21*, *22*). This is especially relevant due to the fact that an unexpected result by the primary care physician could go unnoticed until the following visit. Inclusion of the emergency healthcare services (061 call) as an escalation procedure in the notification protocol entailed a considerable decrease in the number of unsuccessful notifications, especially in the outpatient setting.

It is worth emphasizing that 26.2% of the total number of critical risk results obtained was not notified, under the above mentioned criteria of laboratory professionals. This is of crucial relevance, as a notification excess could minimize the importance of truly urgent notifications (*12*).

Notification failure might entail a delay or lack of diagnosis or treatment, thus having a negative impact in patient’s health, as well as possible legal issues for the healthcare system and for the professionals involved (*23*). The rate of unsuccessful notifications reported by our clinical laboratory meets the acceptability range described elsewhere (0.1-10%) (*24*). In spite of the use of electronic flags for the appraisal, there is, in literature, a considerable number of un-notified critical results, estimated to be up to 10.2% (*25*). Towards a zero-failure aim, a number of tries and period of time need to be established, in order not to alter the normal working of the laboratory. Information regarding the average time to abandonment of communication attempts is sparse but has previously been reported amongst laboratories in United States to be 20.2 minutes for inpatients and 46.3 minutes for outpatients (*7*).

With reference to the use of indicators in our study, it was infeasible to implement some of those recommended by IFCC LEPS (*15*). For instance, the percentage: number of notifications in the established turnaround time (time from result validation until notification)/total number of notifications, as in our protocol there is no specification on turnaround time, and results are validated after notifying them (and not in advance, as suggested by the indicator). However, based on the CLSI GP47 guideline, TAT for routine testing is ‘acceptable’, whereas notifications from the stat laboratory are considered as ‘timely‘, although other sources set the acceptable time between the detection of the critical risk result and its notification between 15-45 minutes (*26*). This observation brings to light the different notification pathways that exist. Although communication is adequate in all of them and in accordance with literature a greater harmonization is still needed and some corrective actions should be established such as timeframes for notification, or avoiding the repetition of critical risk results before reporting them, as repetition of assays has been reported not to contribute to patient safety (*27*-*29*). This could represent a double-edge sword for the patient. On the one hand, it enables the result to be confirmed and errors excluded, whereas, on the other, it delays reporting and increases TAT, which might lead to clinical damage.

In routine biochemistry testing, the magnitudes generating the greatest number of critical results are those related with the most prevalent chronical pathologies in our area, highlighting diabetes mellitus and chronic kidney disease (CKD).

In the ER, a large number of troponin (high-sensitivity troponin I) results are notified successfully, although only the first altered value is notified.

### Satisfaction survey

The results of the questionnaire circulated to all physicians and nurses of our healthcare reveals high satisfaction with the existing protocol in our hospital. Although greater participation would increase the robustness of our conclusions, the 298 answers from professionals represent the total of users of our laboratory services.

Satisfaction was higher in physicians than nurses, maybe due to the fact that this group is responsible for medical actions and has full consciousness of the importance of timely notifications. In addition, primary care professionals gave higher scores, as notifications give to the physician the possibility to anticipate the medical actions which otherwise would occur later at regular medical visit.

### Impact of telephone notifications on medical records

With regard to the indicators for the clinical use of the notification protocol in the ER, as we can state by the revision of medical records, all reviewed critical risk results lead to a corrective action aiming at eradication of the life-threatening condition (*e.g.* resin given to a patient with a potassium concentration of 6.8 mmol/L, or insulin given to a patient with a glucose concentration of 33.5 mmol/L). In contrast, laboratory notifications in the outpatient consultation offices lead to corrective actions in only 54/86 cases (*e.g.* suppression of vitamin D treatment in a patient with calcium concentration of 3.3 mmol/L). Results not leading to a corrective action were mainly from endocrinology (8/32) and nephrology (18/32) outpatients. This observation may be due to the fact that most patients have chronic diseases under treatment, especially individuals with CKD, with a recent kidney transplantation, or unbalanced diabetes. It might be interesting to re-evaluate and redesign the alert list with these medical areas based on our results.

According to the medical records reviewed, all corrective actions were related to the critical result reported and were performed after the notification. However, most of the medical records do not state the laboratory phone call. This fact highlights the challenge of assessing the real clinical impact of the notifications because it is not possible to know specifically whether the notification triggered a corrective action itself, as physicians could have adopted them afterwards, once they had received the electronic laboratory report, or simply based on clinical characteristics (signs, symptoms, *etc.*). The lack of statements on the medical records about laboratory notifications might be due to a lack of time, staff, or the prioritization of clinical actions over the completeness of the medical records.

### Alternative strategies

In our study, a basic approach for the evaluation of the real clinical use of critical result notification by telephone was presented. Despite the inherent difficulties of a proper evaluation of the clinical impact, other possible strategies are possible, including the performance of specific questionnaires, which enable to evaluate the notification protocol to the full extent (quickness of laboratory action, comprehension of the given information and its utility, courtesy, communication style, physician opportunity for timely or early decision making, *etc.*), the acknowledgement in the medical records of the notification made by the laboratory, or the calculation of TAT between telephone notification and the diagnostic/therapeutic action taken by the physician. This places emphasis on the need for healthcare professionals to be more actively involved in the protocol.

This study has several limitations, mainly related to its retrospective nature and trust in the records from the laboratory and hospital information systems. It may be possible that actions and records might not have been performed in a timely manner, and our current system does not allow the true time needed to carry out the notifications to be confirmed. Besides, the influence of haemolysis on the analysis of potassium ion in heparinized whole blood was out of the scope of our study. Regarding the online questionnaire, more detailed and useful data would be obtained if more specific questions were included. In addition to the reduced number of medical records reviewed, the current study design makes it challenging to assess the efficacy of the protocol in terms of clinical impact, so different evaluation approaches need to be adopted.

This study aimed to bring to light the results from our hospital in order to enable further protocols for phone notification of critical laboratory values, hence contributing in the establishment of international harmonized postanalytical phase-related criteria and indicators. According to the results obtained in this study we believe that our protocol of critical risk results is appropriate, since all the indicators measured are in accordance with previous publications, and the satisfaction of physicians and nurses with the current protocol is high. Nevertheless, there are some limitations that hamper the evaluation of the impact on clinical decision making. Alternatives were proposed for a proper and precise evaluation.
